# Intestinal Colonization by *Candida albicans* Alters Inflammatory Responses in Bruton's Tyrosine Kinase-Deficient Mice

**DOI:** 10.1371/journal.pone.0112472

**Published:** 2014-11-07

**Authors:** Karin Strijbis, Ömer H. Yilmaz, Stephanie K. Dougan, Alexandre Esteban, Andrea Gröne, Carol A. Kumamoto, Hidde L. Ploegh

**Affiliations:** 1 Whitehead Institute for Biomedical Research, Cambridge, Massachusetts, United States of America; 2 Department of Pathobiology, Faculty of Veterinary Medicine, Utrecht University, Utrecht, The Netherlands; 3 Department of Molecular Biology and Microbiology, School of Medicine, Tufts University, Boston, Massachusetts, United States of America; University of Wisconsin Medical School, United States of America

## Abstract

The commensal yeast *Candida albicans* is part of the human intestinal microflora and is considered a “pathobiont”, a resident microbe with pathogenic potential yet harmless under normal conditions. The aim of this study was to investigate the effect of *C. albicans* on inflammation of the intestinal tract and the role of Bruton's tyrosine kinase (Btk). Btk is an enzyme that modulates downstream signaling of multiple receptors involved in innate and adaptive immunity, including the major anti-fungal receptor Dectin-1. Colitis was induced in wild type and Btk-/- mice by treatment with dextran sodium sulfate (DSS) and the gastrointestinal tract of selected treatment groups were then colonized with *C. albicans*. Colonization by *C. albicans* neither dampened nor exacerbated inflammation in wild type mice, but colon length and spleen weight were improved in Btk-deficient mice colonized with *C. albicans*. Neutrophil infiltration was comparable between wild type and Btk-/- mice, but the knockout mice displayed severely reduced numbers of macrophages in the colon during both DSS and DSS/*Candida* treatment. Smaller numbers and reduced responsiveness of Btk-/- macrophages might partially explain the improved colon length of Btk-/- mice as a result of *Candida* colonization. Surprisingly, DSS/*Candida*-treated Btk-/- animals had higher levels of certain pro-inflammatory cytokines and levels of the anti-inflammatory cytokine TGF-β were reduced compared to wild type. A clustering and correlation analysis showed that for wild type animals, spleen TGF-β and colon IL-10 and for Btk-/- spleen and colon levels of IL-17A best correlated with the inflammatory parameters. We conclude that in Btk-/- immunocompromised animals, colonization of the gastrointestinal tract by the commensal yeast *C. albicans* alters inflammatory symptoms associated with colitis.

## Introduction

The intestinal microflora plays an important not only in establishing immune tolerance but also in the development of inflammatory bowel disease (IBD) and obesity. While studies of the microbiome have mostly focused on commensal bacteria, several species of fungi are also major constituents of the mammalian gastrointestinal system, with highest fungal concentrations in the colon [1], [2]. It is estimated that fungi are detectable in all gastrointestinal segments of about 70% of healthy adults [3], with *Candida* spp. being predominant [4], [5]. *Candida albicans* is a commensal fungus of the human gastro-intestinal tract, capable of causing life-threatening opportunistic fungal infections [6]. *C. albicans* is considered opportunistic pathogen or a “pathobiont”, a resident microbe with pathogenic potential yet harmless under normal conditions. Gut-colonizing *Candida* can cause candidaemia [7], [8], but mucosal damage and neutropenia are required for *Candida* dissemination from the colon [9]. An increase in fungal load and *Candida* species was observed in patients suffering from Crohn's disease [10].

The C-type lectin Dectin-1, which recognizes cell wall β1,3-glucan, is the major receptor involved in antifungal immune responses [11]. While essential during antifungal immune responses during systemic *C. albicans* infections in mice, lack of Dectin-1 does not affect gastrointestinal colonization by *C. albicans* [12]. Dectin-1 knockout mice are more sensitive to chemically-induced colitis, presumably due to diminished immune surveillance of endogenous fungal species [1]. The contribution of fungi to the development of IBD is further illustrated by human polymorphisms in Dectin-1 associated with ulcerative colitis (UC) and its signaling molecule CARD9. The latter has been associated with both UC and Crohn's disease [1], [13], [14]. Serum antibodies against yeast cell wall components (anti-glucan, anti-laminarin and anti-chitin antibodies) are predictive for Crohn's disease and ulcerative colitis [15].

We previously identified Bruton's tyrosine kinase (Btk) as a signaling molecule in the Dectin-1-mediated immune response to *C. albicans* [16], where it contributes to macrophage phagocytosis and cytokine production. Btk-/- mice are more susceptible to systemic *C. albicans* infections. Btk also modulates downstream signaling of other immune receptors, including the Fcγ receptor [17] and the B-cell receptor, leading to reduced B cell numbers and antibody secretion [18], [19]. For this study, we investigated the role of Btk during gastrointestinal colonization with *C. albicans*. Our findings add to a growing body of work that indicates probiotic effects of commensal yeasts in the gastrointestinal tract.

## Materials and Methods

### *Candida* cultures

*C. albicans* strain SC5314 was cultured in standard YPD medium (2% bactopeptone, 1% yeast extract, 2% glucose and 80 µg/ml uridine) for 16 hours at 30°C in a shaker.

### Mouse models of *Candida* colonization

Animals used in this study were housed at the Whitehead Institute for Biomedical Research or at Tufts University, which are both certified by the United States Office of Laboratory Animal Welfare (OLAW) under the guidance of the Public Health Service (PHS) Policy on Humane Care and Use of Laboratory Animals. All experiments were done in compliance with regulatory guidelines defined by the Tufts University IACUC committee and Whitehead Institute IACUC committee and in accordance with procedures approved by the Massachusetts Institute of Technology Committee on Animal Care (Ploegh lab, CAC# 1011-123-14). Btk-/- animals were a kind gift from Whasif Khan [18]. For the Dectin-1 staining experiments, antibiotic-treated mice were colonized with *C. albicans* as described previously [20]. In short, 5–7 week-old BALB/c mice were treated with antibiotics (tetracycline, 1 mg/ml; streptomycin, 2 mg/ml; gentamicin, 0.1 mg/ml) in the drinking water and fed the purified diet AIN-93G containing glucose [21]. This diet was used because numerous yeast-form particles are found in the cecum contents of mice fed yeast extract-containing chow. Mice were inoculated with 0.1 ml *C. albicans* strain CKY101 and sacrificed at 4 and 18 days post-inoculation to obtain cecal contents for microscopy analysis [22]. Cecum contents was enriched for microbial cells by purification on Percoll gradients and stained with the Dectin1-CRD-Alexa^647^ probe [16]. For the DSS experiments, age-matched C57BL/6 mice were used as wild type control animals. To minimize differences in microbiota composition, all mice were bred and maintained in the same room at the Whitehead Animal Facility. All mice were 4 months old at the onset of experiments. Both males and females were used and genders were matched between wild type and Btk-/- groups. Chemically-induced colitis and *Candida* colonization experiments were performed as previously described [23], [24]. In short, colitis was experimentally induced by administration of 5% Dextran sodium sulfate (DSS) in the drinking water from day 1. On day 3, mice were inoculated with *C. albicans* strain SC5314 by oral gavage with 1×10^7^ colony forming units (CFU) in a final volume of 200 µl PBS. Mice were weighed and monitored daily and the experiment was terminated when animals had lost >20% of initial body weight. DSS was continuously administered until sacrifice. Colon and cecum were harvested for determination of colon length, cytokine levels and histology and cecum content for enumeration of fungal burdens. Spleens were harvested to determine weight and cytokine levels. To determine fungal burden, cecum contents were homogenized and plated on YPD plates with antibiotics.

### Histology

For histology, tissues were fixed in 10% formalin in PBS, embedded in paraffin, sectioned and stained with Hematoxylin and Eosin (H&E) or Gomori Methenamine Silver (GMS). Immunohistochemistry was performed with antibodies directed against Myeloperoxidase (MPO) to detect neutrophils or CD68 to detect macrophages. For MPO staining, antigen retrieval was performed in Biocare's Pressure Cookers using EDTA, pH 8 solution, a polyclonal rabbit anti-human myeloperoxidase primary antibody (Dako) was diluted to 1∶3500 and a Dako's Rabbit Envision detection kit was used. For CD68 staining antigen retrieval was performed with Borg Decloaker RTU solution (Biocare Medical) in a pressurized Decloaking Chamber (Biocare Medical) for 3 min. As a primary antibody rat monoclonal anti-CD68 [FA-11] antibody (Abcam) was diluted to 1∶500 followed by biotin-conjugated secondary donkey anti-rat antibodies (Jackson ImmunoResearch). The Vectastain Elite ABC immunoperoxidase detection kit (Vector Labs PK-6101) followed by Dako Liquid DAB1 Substrate (Dako) was used for visualization. For quantification of neutrophil and macrophage numbers, 5×100 grids were counted at a 40x magnification for 3 individual animals.

### Cytokine measurements

Colons and spleen were homogenized in 1 ml PBS followed by cytokine analysis using a Th1/Th2/Th17 mouse cytometric bead array (BD Biosciences) and LSR Flow Cytometer (Whitehead Institute Flow Cytometry Core) according to the manufacturer's directions. Separate ELISA assays were performed for IL-1β (BD Biosciences) and TGF-β (Abcam) according to the manufacturer's instructions.

### Statistical analysis

For regular statistical analysis between samples within groups a one-way ANOVA test was performed with Holm-Sidak correction for multiple comparisons using Prism 6 software. We applied this test to 7 comparisons for all our experiments (colon length, spleen weight and cytokine data). 7 comparisons were: 1) wild type control vs wild type DSS. 2) wild type control vs wild type DSS/*Candida*. 3) wild type DSS vs wild type DSS/*Candida*. 4) Btk-/- control vs Btk-/- DSS. 5) Btk-/- control vs Btk-/- DSS/*Candida*. 6) Btk-/- DSS vs Btk-/- DSS/*Candida*. 7) wild type DSS/*Candida* vs Btk-/- DSS/*Candida.*

A student t test was used for a comparison between two values using Prism 6 software. For the clustering analysis, uncentered correlation with average linkage was performed using the program Cluster 3.0 [25]. Clustered matrices were visualized with Java TreeView [26]. Linear regression analysis was performed using R software (http://www.r-project.org/) to identify potential correlations between cytokine levels and colon lengths or spleen weights.

## Results

### *C. albicans* adapts to the gastrointestinal tract by formation of hyphae and exposure of β1,3-glucan

**Figure 1:**
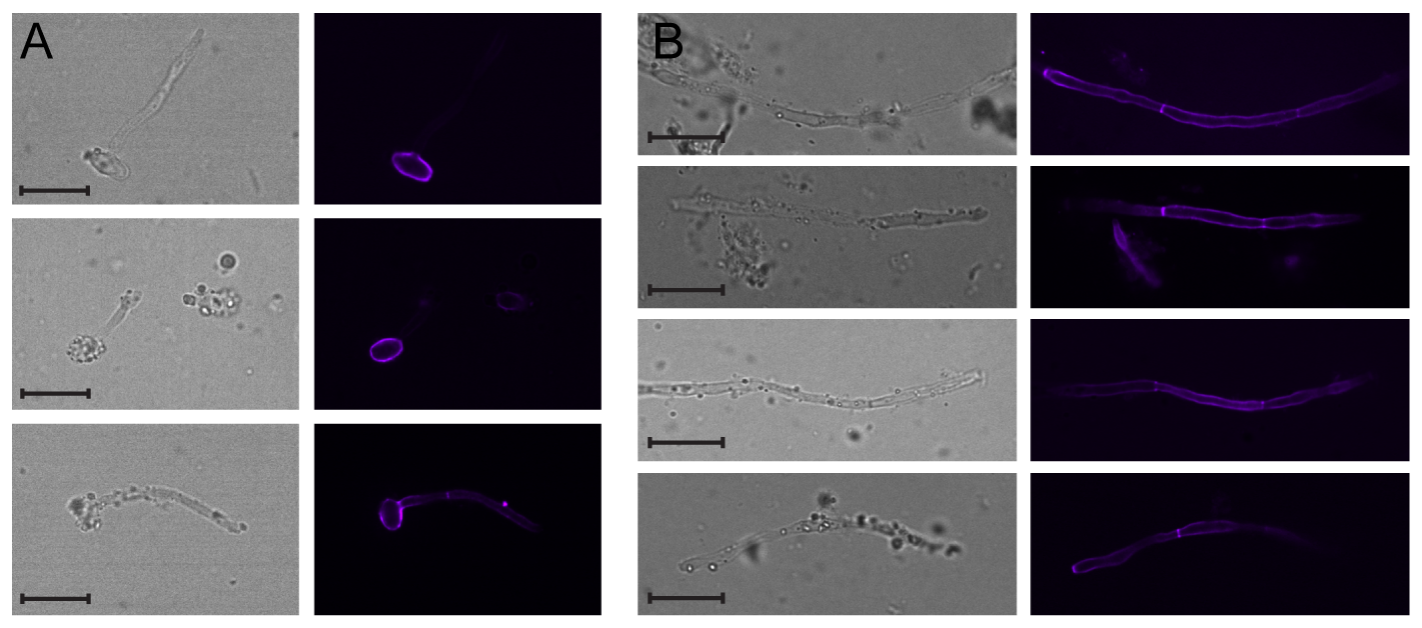
Morphology and β1,3-glucan exposure of *C. albicans* adapted to the gastrointestinal tract. A: *C. albicans* isolated from mouse cecum 4 days after inoculation and stained with Dectin1-CRD-Alexa^647^. After 4 days of gastrointestinal adaptation, the majority of *C. albicans* display short (pseudo)hyphal morphology in which β1,3-glucan exposure is limimited. B: *C. albicans* isolated from cecum 18 days after inoculation and stained with Dectin1-CRD-Alexa^647^. The majority of *C. albicans* display short (pseudo)hyphal morphology. After 18 days of gastrointestinal adaptation, the majority of *C. albicans* display long hyphal morphology with extensive β1,3-glucan exposure. Experiments were performed twice with two animals in each group, representative images are shown.

We first studied the adaptation of *C. albicans* to the gastrointestinal tract and the exposure of the Dectin-1 ligand β1,3-glucan in the yeast cell wall. To allow for colonization of the gastrointestinal tract in the absence of inflammation, we chose the previously described mouse model of *C. albicans* inoculation by oral gavage after antibiotics treatment [20]. Four or 18 days post-inoculation, *C. albicans* was retrieved from the caecum and stained with a fluorescent soluble carbohydrate recognition domain of Dectin-1 (Dectin1-CRD-Alexa647) [16]. At the 4-day time point, yeast morphology presented as short hyphae emanating from the yeast particles. The yeast-form mother cell stained extensively with Dectin1-CRD-Alexa647, while the germ tube showed less staining (**Figure 1A**). At the 18-day time point, we observed long hyphae that showed extensive staining with the soluble receptor domain (**Figure 1B**). We conclude that after an initial phase of adaptation to the gastrointestinal tract, *C. albicans* filaments contain β1,3-glucan that is accessible to Dectin-1.

### Gastrointestinal colonization by *C. albicans* has protective effects in Btk-/- animals

**Figure 2:**
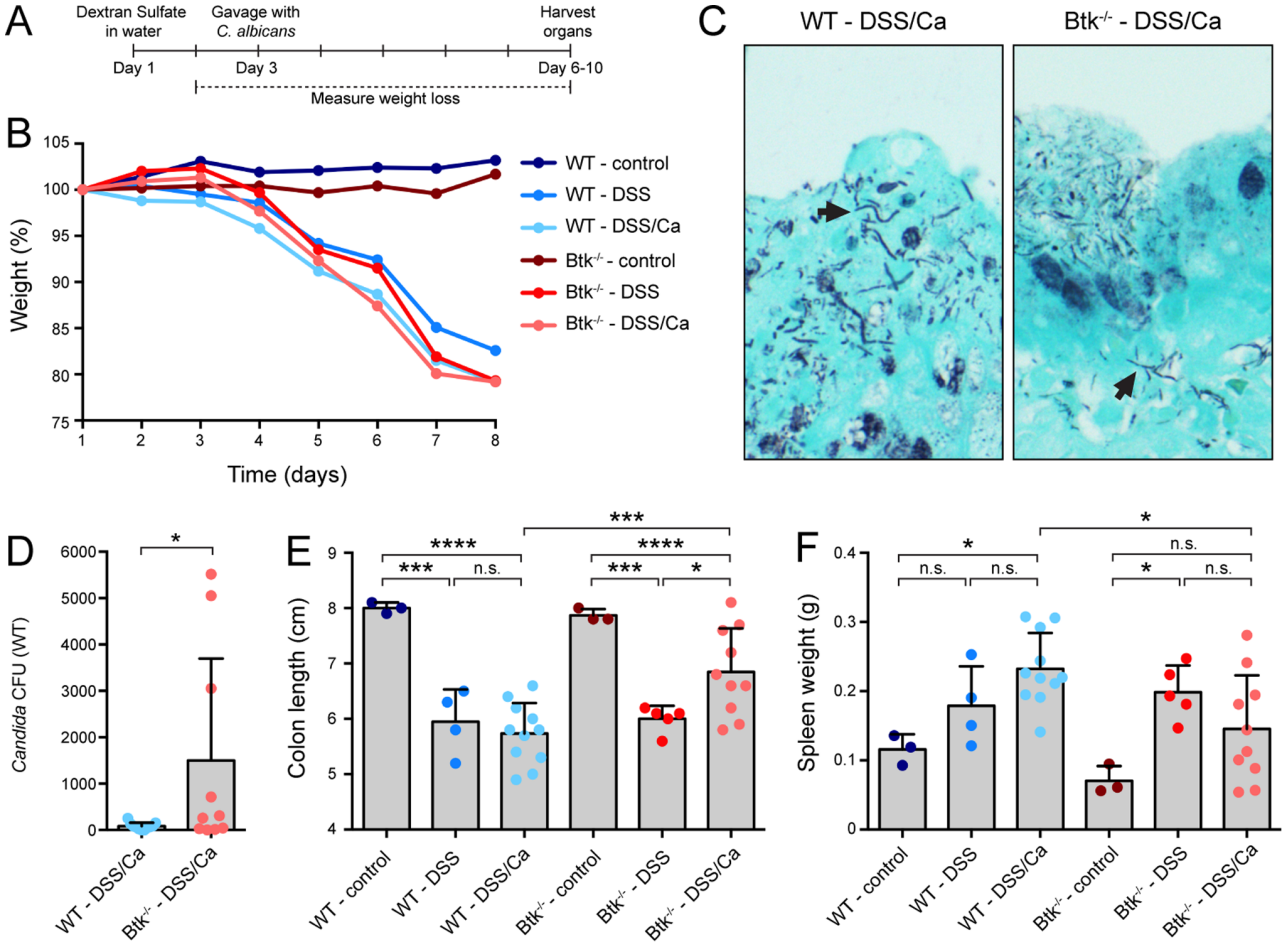
Colonization by *C. albicans* reduces DSS-induced inflammation in Btk-/- mice. A: Experimental design of DSS-induced colitis in combination with *C. albicans* colonization of the gastrointestinal tract. DSS was administered in the drinking water starting on day 1. On day 3, 1×10^7^ CFU of *Candida albicans* was administered to the DSS/*Candida* treatment groups by oral gavage. Control groups included 3, DSS groups 5 and DSS/*Candida* groups included 10-11 animals. Animals were sacrificed on day 8 when the first animals reached 80% of initial body weight. B: Weight loss curve of the indicated groups of mice during DSS- or DSS/*Candida* treatment. C: Gomori Methenamine Silver (GMS) staining of wild type and Btk-/- colon after DSS/*Candida* treatment. Arrows indicate *Candida* hyphae. D: *C. albicans* colony forming units (CFUs) in cecum content in wild type and Btk-/- mice during DSS/*Candida* treatment. E: Spleen weights of all mice and treatment groups in DSS/*Candida* experiment. F: Colon lengths of all mice and treatment groups in DSS/*Candida* experiment. All graphs show individual data points, the means of the indicated groups and error bars depict standard deviations. *: p<0.05, **: p<0.01, ***: p<0.005, ****: p<0.001.

Next we investigated the impact of *C. albicans* colonization on inflammation of the gastrointestinal tract. We chose the dextran sodium sulfate (DSS) colitis mouse model in conjunction with *C. albicans* colonization by oral gavage [23], [24]. Colitis was induced in wild type and Btk-/- animals by provision of DSS in the drinking water, followed by an oral inoculum of 1×10^7^ colony forming units of *C. albicans* on day 3 (**Figure 2A**). The experiment included control, DSS only and DSS/*Candida* treatment groups of wild type and Btk-/- animals. Animals were sacrificed 8 days after initiation of the experiment. DSS-treated and DSS/*Candida*-treated wild type and Btk-/- animals lost weight at a similar rate (**Figure 2B**). For both wild type and Btk-/- animals, *Candida* hyphae were visible when colons were stained with fungi-specific Gomori Methenamine Silver (GMS) (**Figure 2C**). The hyphal morphology observed in these histology slides corroborates our results that *C. albicans* adapts to the gastrointestinal tract by hyphae formation (**Figure 1**). We next quantified *C. albicans* colonization of the wild type and Btk-/- DSS/*Candida* groups. The numbers of *Candida* colony-forming units (CFUs) from caecum samples were slightly higher in the DSS/*Candida*-treated Btk-/- animals compared to wild type (**Figure 2D**). Addition of DSS to the drinking water resulted in statistically significant shortening of the colon of wild type animals (**Figure 2E**). Wild type animals that received the DSS/*Candida* combination treatment also showed significantly reduced colon lengths compared to the wild type control group. There was no difference in colon length between the wild type DSS and DSS/*Candida*-treated groups, suggesting that *C. albicans* neither increased nor decreased inflammation. Colon shortening as a result of DSS-only treatment was also observed for the Btk-/- mice. However, the DSS/*Candida*-treated Btk-/- animals displayed significantly longer colons compared to DSS-treated Btk-/- mice (p<0.05) and compared to DSS/*Candida*-treated wild type animals (p<0.005) (**Figure 2E**). These results suggest that in the Btk-/- animals, colonization by *C. albicans* may dampen inflammation. As a proxy for systemic inflammation we measured spleen weight, as increased leukocyte numbers enlarge the spleen. Wild type animals showed a significant increase in spleen weight upon treatment with the DSS/*Candida* combination (**Figure 2F**). Btk-/- animals treated with DSS had significantly enlarged spleens and spleen weight was reduced in the DSS/*Candida*-treated group, although this difference did not reach statistical significance. DSS/*Candida*-treated Btk-/- animals showed reduced spleen weight compared to the wild type DSS/*Candida*-treated animals (p<0.05). *Candida* colonization thus affords a measure of protection against DSS-induced colitis in Btk-/- animals.

### Colons of Btk-/- animals have reduced macrophage infiltrates

**Figure 3:**
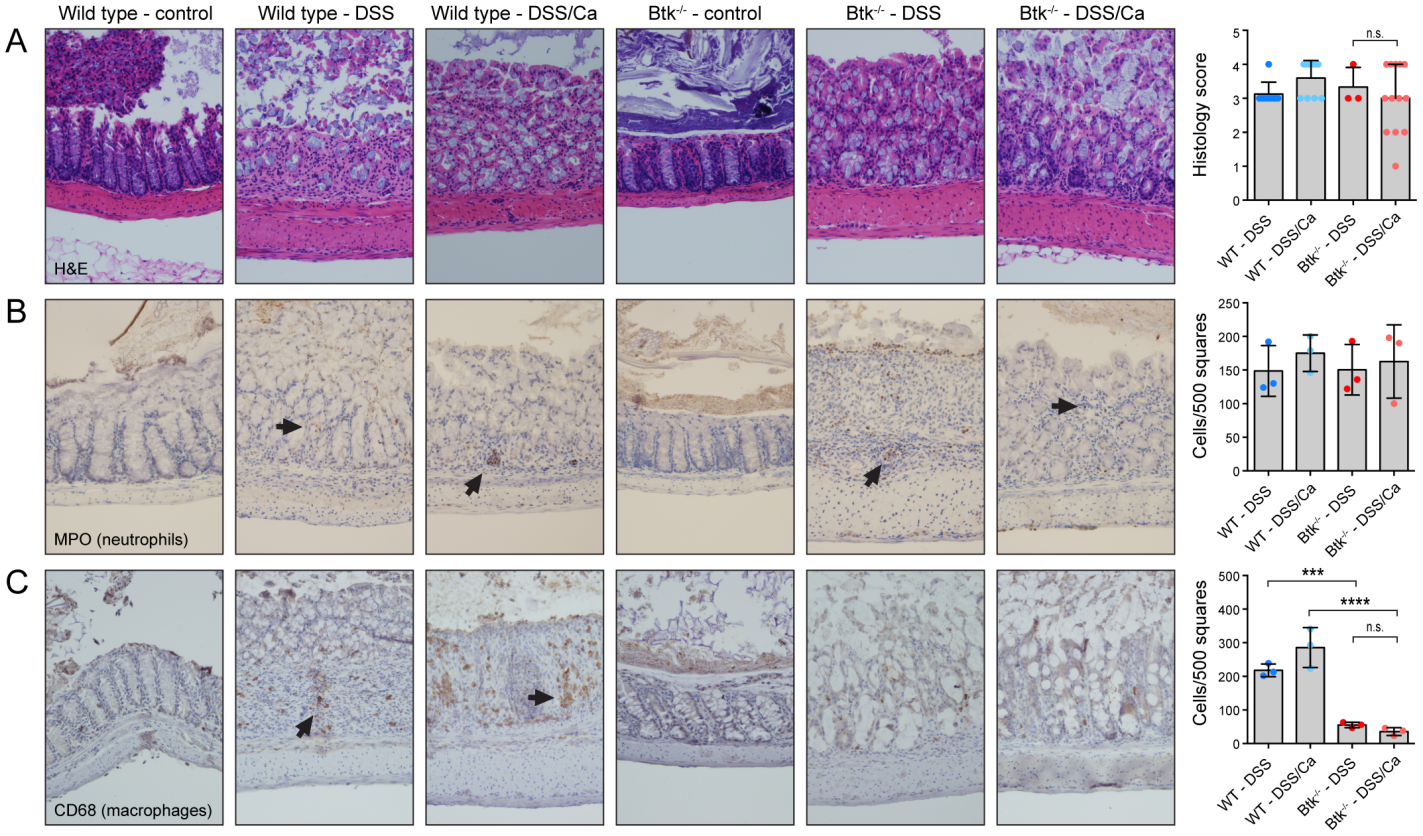
Btk-/- animals have reduced colonic macrophages, but not neutrophils. Mice were treated with DSS or the DSS/*Candida* combination as described in the legend of Figure 2. Sample size (number of animals): control groups (1), DSS groups (3), DSS/*Candida* groups (3). Animals were sacrificed on day 8 and the distal colons were fixed and prepared for histology. A: H&E staining of indicated mouse distal colon sections. Right: H&E stainings were blindly graded based on the following scheme: 1. Normal mucosa. 2. Cryptitis. 3. Crypt abscesses. 4. Focal ulcerations. 5. Multifocal ulcerations. B: Immunohistochemical staining using MPO antibody shows neutrophil infiltration in the indicated mouse distal colon sections. Right: Quantification of infiltrating neutrophils in DSS and DSS/*Candida* conditions. C: Immunohistochemical analysis with CD68 antibody showing macrophages in the indicated mouse distal colon sections. Right: Quantification of macrophages in DSS and DSS/*Candida* conditions. ***: p<0.005, ****: p<0.001. Representative pictures of each genotype and staining are shown.

To further assess the severity of inflammation, we performed H&E staining of the distal colon of the wild type and Btk-/- control and treatment groups. Microscopy showed that all treatment groups suffered from severe cryptitis, crypt abscesses and (multi)focal ulcerations (**Figure 3A**). For a quantitative analysis, the H&E-stained colons were blindly graded for the presence of normal mucosa (1), cryptitis (2), crypt abscesses (3), focal ulcerations (4) and multifocal ulcerations (5). The colons of some Btk-/- animals of the DSS/*Candida* treatment groups showed improved histology, but the difference between groups did not reach statistical significance. To examine the composition of the colon inflammatory infiltrates we performed immunohistochemical analysis. Neutrophil invasion as measured by MPO staining was observed upon treatment with DSS and DSS/*Candida* in both wild type and Btk-/- colons and the number of neutrophilic infiltrates was comparable between groups (**Figure 3B**). A CD68 stain showed macrophage infiltrates in the wild type DSS and DSS/*Candida* condition, but a severely reduced number of macrophages in the colons of Btk-/- animals (**Figure 3C**).

### Btk-/- animals show elevated pro-inflammatory cytokines and decreased TGF-β levels

**Figure 4:**
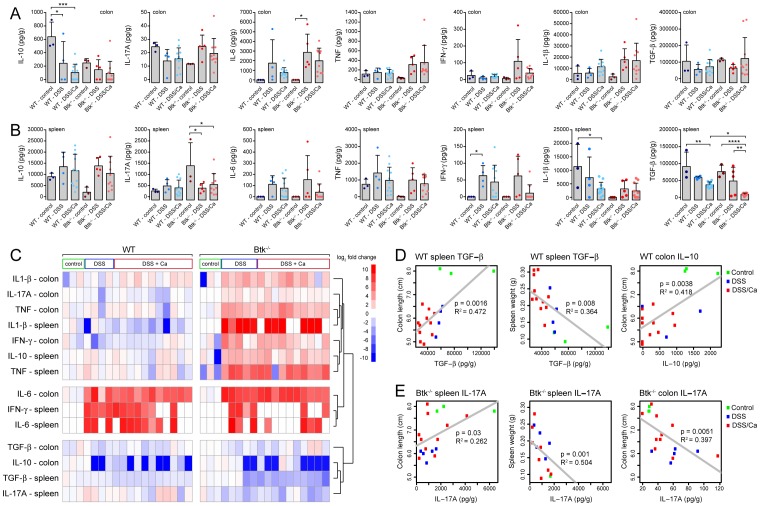
TGF-β, IL-10 and IL17A in colon and spleen are correlated with inflammatory parameters. Mice were treated with DSS or the DSS/*Candida* combination as described in the legend of Figure 2. Sample size (number of animals): control groups (3), DSS groups (5), DSS/*Candida* groups (10-11). Cytokine levels were determined in lysates of spleen and proximal colon. IL10, IL-17A, IL-6, TNF, IFN-γ, IL-1β and TGF-β cytokine levels in spleen lysates (A) and lysates of the proximal colon (B). Bars and error bars show the mean and standard deviation of the indicated groups and data points resembling individual mice are plotted. A one-way ANOVA test with Holm-Sidak correction for multiple comparisons was applied to determine statistical significance. *: p<0.05, **: p<0.01, ***: p<0.005, ****: p<0.001, C: Clustering analysis of all cytokine data. Each vertical column represents one animal and its respective cytokine values. The average value of the wild type and Btk-/- control groups was set at 0 and DSS and DSS/*Candida* data were expressed as relative log values. Color codes of relative log values ranging from -10 to 10 are indicated in the legend. D, E: Significantly correlated data after Pearson analysis of cytokine data versus colon length or spleen weight. For wild type animals, spleen TGF-β correlated with colon length and spleen weight and colon IL-10 correlated with colon length (D). For Btk-/- animals, spleen IL-17A correlated with both colon length and spleen weight, while colon IL-17A was correlated with colon length (E).

We next investigated if colonization by *C. albicans* and the reduced macrophage numbers in the Btk-/- colons impact local cytokine levels in the colon and systemic cytokine levels in the spleen. Cytokine measurements were performed on lysates of the proximal colon (**Figure 4A**) and spleen (**Figure 4B**). Statistical differences between 7 selected groups were determined by one-way ANOVA test with Holm-Sidak's correction for multiple comparisons. In the colon of wild type animals, IL-10 levels were significantly lowered as a result of DSS or DSS/*Candida* treatment (p<0.05 and p<0.005, respectively) (**Figure 4A**). Colonic IL-6 was elevated in all wild type and Btk-/- treatment groups, but only the difference between Btk-/- control and DSS treatment was significant (p<0.05) (**Figure 4A**). In the spleens of Btk-/- mice, IL-17A levels were significantly decreased in both treatment conditions (p<0.05) (**Figure 4B**). This decrease is due mainly to the high levels of IL-17A in Btk-/- control spleens, suggesting elevated levels of this cytokine in the absence of any treatment or inflammation. Furthermore, IFN-γ levels in the spleen were significantly higher in DSS-treated wild type mice compared to the control group. Surprisingly, wild type spleen IL-1β levels were lowered in response to DSS/*Candida* treatment, while Btk-/- animals showed a reverse trend. Of all cytokines analyzed, spleen TGF-β levels showed the most striking differences between treatment groups. TGF-β levels were similar in wild type and Btk-/- control groups and DSS/*Candida* treatment reduced TGF-β levels for both genotypes (p<0.01 and p<0.001, respectively). In Btk-/- animals, *C. albicans* colonization in combination with DSS treatment led to a significant decrease in TGF-β levels compared to DSS treatment alone (p<0.01). To investigate functional relationships between the investigated cytokines we performed clustering analysis of all cytokine measurements. The values of the wild type and Btk-/- control groups were averaged at 0 and other measurements were expressed as relative log values (**Figure 4C**). The first functional cluster of pro-inflammatory cytokines that emerged consisted of colon IL-1β, colon IL-17A, colon TNF, spleen IL-1β, colon IFN-γ, spleen IL-10 and spleen TNF. For this group of cytokines, a general upregulation was observed in the DSS and DSS/*Candida* conditions, which was more pronounced in the Btk-/- groups. A second cluster of pro-inflammatory cytokines consisted of colon IL-6, spleen IFN-γ and spleen IL-6 and was equally elevated in DSS and DSS/*Candida* groups of both wild type and Btk-/- animals. A third cluster of anti-inflammatory cytokines consisting of colon TGF-β, colon IL-10, spleen TGF-β and spleen IL-17A was downregulated in the DSS and DSS/*Candida* groups of both wild type and Btk-/- animals. In summary, Btk-/- treatment groups display a general increase in pro-inflammatory cytokine levels in colon and spleen and reduced levels of splenic TGF-β compared to wild type animals.

### TGF-β, IL-10 and IL-17A are associated with inflammation

Because of the reduced inflammation seen in the Btk-/- animals, we were curious which of the cytokine profile best correlated with colon length or spleen weight in wild type and Btk-/- animals. We performed linear regression analysis and determined p and R^2^ values of the correlation coefficients. For the wild type animals, TGF-β levels correlated with colon length and negatively correlated with spleen weight (**Figure 4D**). Colon IL-10 levels and colon length were also significantly correlated. In other words, lowering of the anti-inflammatory cytokines TGF-β and IL-10 best predicts inflammation in wild type animals. For the Btk knockout animals, spleen IL-17A correlated with colon length and negatively correlated with spleen weight, while colon IL-17A was negatively correlated with colon length (**Figure 4E**). We conclude that in Btk-/- animals, reduced inflammation is associated with high spleen IL-17A levels and decreased IL-17A in the colon.

## Discussion

*C. albicans* is a commensal yeast that is part of the normal human microflora, although most studies have focused on the pathogenic properties of this opportunistic fungus. Here we show that colonization of the gastrointestinal tract by *C. albicans* alters inflammatory responses in a mouse model of colitis and improves some colitis parameters. Whereas wild type animals do not noticeably benefit (or suffer) from the presence of *C. albicans*, Btk-/- animals show a measure of protection as assessed by colon length and spleen weight (**Figure 2**). Macrophage, but not neutrophil, infiltration of the colon in response to DSS or DSS/*Candida* treatment is severely reduced in Btk-/- animals (**Figure 3**). We hypothesize that this reduction in combination with the lowered phagocytic capacity and reduced ROS production of Btk-/- macrophages causes the altered inflammatory responses of the Btk-/- animals during *C. albicans* colonization. In the colons and spleen of Btk-/- animals we find elevated levels of pro-inflammatory cytokines and reduced levels of anti-inflammatory cytokines during DSS or DSS/*Candida* treatment. For wild type animals, a reduction in colon IL-10 and spleen TGF-β is associated with increased inflammation. For Btk-/- animals, reduced inflammation is associated with elevated spleen IL-17A levels and reduced colon IL-17A levels (**Figure 4**).

We demonstrate that *Candida* adapts to the gastrointestinal tract by the formation of hyphae and exposure of the Dectin-1 ligand β1,3-glucan (**Figure 1A and B**). *C. albicans*, while in the gut, can therefore have potent immuno-stimulatory effects. Previous studies showed staining of fungal cells using a Dectin-1 CRD domain, but the identity and source of the stained cells was unknown [1]. In our experiments, mice were fed on feed devoid of yeast extract, thereby excluding an extraneous source of yeast-like particles. The combination of staining with the fluorescent Dectin-1 CRD domain allowed us to identify gut-adapted *C. albicans* in murine cecal contents. At 4 days post-inoculation, germ tubes produced by *C. albicans* cells colonizing the gastrointestinal tract stained poorly with Dectin-1, consistent with previous observations of differential exposure of β1,3-glucan in yeasts and hyphal elements grown in laboratory culture [27]. Later in the time course of colonization (day 18 post-inoculation), gene expression in colonizing cells changes [28] and we found numerous hyphal filaments that stained with Dectin-1. The observation of high β1,3-glucan exposure late during colonization replicates earlier findings of β1,3-glucan unmasking late during systemic infection [29]. Physiological changes that affect Dectin-1 binding occur as *C. albicans* adapts to growth in the gastrointestinal tract.

Conflicting data have been published on the importance of Dectin-1 during *Candida* colonization or the development of colitis. Dectin-1 knockout mice were found to be equally sensitive [30] or more susceptible to DSS-induced colitis [1]. The latter report points at the role of endogenous fungi of the gastrointestinal tract, therefore a different composition of the microbiome may explain the opposing results. However, in our hands the colon length of the DSS/*Candida*-treated Dectin1-/- animals was not significantly different from DSS/*Candida*-treated wild type animals (data not shown). In DSS-independent experiments, colonization of the gastrointestinal tract by *C. albicans* was not affected by the presence or absence of Dectin-1 [12]. Together, these results suggest that Dectin-1 plays a role in anti-fungal immunity only in conjunction with a diseased state of the colon. Besides Dectin-1, pattern recognition receptors like DC-SIGN, Mannose receptor and Mincle are known to be involved in cellular immune responses against *C. albicans*. Their roles during *C. albicans* colonization of the gastrointestinal tract remain to be investigated.

Which of the changes that result from the absence of Btk could explain the observed protective effect of the DSS/*Candida* treatment? Btk is an important component of B-cell receptor signaling and Btk-/- animals have reduced B cell numbers and impaired antibody secretion [18], [19]. B cells are prominent immune cells in the small intestine's lamina propria where their ability to secrete IgA is crucial for normal intestinal gene expression [31]. Despite this, DSS induces colitis in B cell-deficient mice [32] without affecting composition of the gastrointestinal microbiome [31]. In addition to B cell defects, Btk deletion also leads to innate immune deficiencies. Btk-/- mice have reduced numbers of polymorphonuclear leukocyte (PMN) numbers and Btk-/- neutrophils are deficient in ROS and NO production [33]–[35]. Mangla *et al*. also reported reduced inflammatory responses of Btk-/- knockout mice in different infection models [33].

Our immunohistochemistry experiments show reduced numbers of macrophages in DSS- and DSS/*Candida*-treated Btk-/- colons while neutrophil numbers are comparable to wild type (**Figure 3**). Because infiltrating macrophages and neutrophils cause severe immunopathology during colitis, we hypothesize that their lower numbers and reduced reactivity contribute to the reduced inflammation in the Btk-/- DSS/*Candida* treatment group. We previously showed that Btk interacts with Dectin-1 and that Btk-/- macrophages have phagocytic defects [16], possibly leading to an increase in fungal load in Btk-/- animals (**Figure 2D**). In conjunction with the lower numbers of macrophage infiltrates in Btk-/- colons, these observations might explain the altered inflammatory response of Btk-/- animals in response to DSS treatment and *C. albicans* colonization.

Cytokine analysis of spleen samples showed a general pro-inflammatory response with upregulation of IL1-β, IL-17A (colon), TNF, IFN-γ, IL-10 (spleen) and IL-6) and down regulation of TGF-β, IL-10 (colon), IL17A (spleen) (**Figure 3A, B, C**). There was no clear-cut effect of *Candida* colonization on the cytokines measured. Clustering analysis showed that wild type and Btk-/- animals treated with DSS or DSS/*Candida* similarly induce expression of IFN-γ and IL-6, cytokines often associated with trauma induced by *C. albicans* infection/colonization (**Figure 3C**). However, Btk-/- animals treated with DSS or DSS/*Candida* showed a higher upregulation of IL1-β, IL-17A, TNF, IFN-γ and IL-10 compared to wild type treatment groups. Therefore we conclude that reduced inflammatory responses in Btk-/- animals during *Candida* colonization are associated with relatively high levels of pro-inflammatory cytokines. These observations support the hypothesis that reduced inflammatory responses in Btk-/- animals are due to less responsive innate immune cells.

Correlation analysis showed that inflammation in wild type mice is associated with decreased TGF-β and IL-10 levels (**Figure 4D**). TGF-β is an important anti-inflammatory cytokine that is involved in the induction of regulatory T cells and Th17 cells. Interestingly, it has been previously reported that TGF-β transgenic mice develop age-related autoimmune disease, characterized by T-cell activation and colitis [36]–[38]. The anti-inflammatory IL-10 is produced by monocytes and epithelial cells and plays an important role in immune regulation in relation to microbiota in the gastrointestinal tract [39]. Both TGF-β and IL-10 are important cytokines in maintenance of gut health, which fits with the negative correlation with DSS-induced inflammation in wild type animals. The cytokine that is most closely correlated with the inflammatory response of Btk-/- animals is IL-17A. Increased spleen IL-17A levels are associated with increased colon length and decreased spleen weight. On the contrary, low colon IL-17A levels are associated with increased colon length (**Figure 4E**). In other words, “healthy” Btk-/- animals have high spleen IL-17A levels and low colon IL-17A levels. When comparing IL-17A levels in control groups and DSS and DSS/*Candida* groups, Btk-/- animals show an opposite trend compared to wild type animals. In Btk-/- animals, colon IL-17A is upregulated during inflammation and in the spleen lower levels of IL-17A are observed (**Figure 4A, B**). IL-17A is a key cytokine in the Th17 anti-fungal immune response that can be produced by Th17 cells [40], [41], but also to a minor extend by neutrophils [42] and innate lymphoid cells [43]. Th17 cytokines including IL-17A are generally linked to severity of colitis but also induce protective regulatory mechanisms in epithelial cells [44].

Considering it's central role in the development of colitis, altered regulation and secretion of IL-17A might contribute to the reduced inflammatory response in Btk-/- animals.

Favorable effects of *C. albicans* colonization of the gut were previously reported. β-glucan, a major *C. albicans* cell wall component and the ligand Dectin-1, was shown to have potent anti-inflammatory effects in a mouse colitis model [45]. In addition, in the recovery phase after antibiotics treatment, the presence of *C. albicans* promoted bacterial diversity in the mouse caecum, specifically enriching *Bacteroidetes* populations and reducing *Lactobacillus* species [46], [47]. This link between *C. albicans* and Bacteroidetes is interesting, because *Bacteroides fragilis* was shown to induce Tregs and mucosal tolerance [48]. The pleiotropic effect of Btk deletion makes it difficult to pinpoint the mechanism of protection in the DSS/*Candida*-treated Btk-/- animals. Conditional knockout animals with Btk deletion in specific cell types might assist in future experiments. In addition, the available Btk inhibitors in combination with *Candida* colonization might replicate the protective phenotype and thus Btk could be a possible target for the treatment of inflammatory conditions such as ulcerative colitis and Crohn's disease.

## References

[1] Iliev ID, Funari VA, Taylor KD, Nguyen Q, Reyes CN, et al (2012) Interactions between commensal fungi and the C-type lectin receptor Dectin-1 influence colitis. Science 336: 1314–1317. 22674328 10.1126/science.1221789 PMC3432565

[2] Ghannoum MA, Jurevic RJ, Mukherjee PK, Cui F, Sikaroodi M, et al (2010) Characterization of the oral fungal microbiome (mycobiome) in healthy individuals. PLoS Pathog 6: e1000713. 20072605 10.1371/journal.ppat.1000713 PMC2795202

[3] Schulze J, Sonnenborn U (2009) Yeasts in the gut: from commensals to infectious agents. Dtsch Arztebl Int 106: 837–842. 20062581 10.3238/arztebl.2009.0837 PMC2803610

[4] Ott SJ, Kuhbacher T, Musfeldt M, Rosenstiel P, Hellmig S, et al (2008) Fungi and inflammatory bowel diseases: Alterations of composition and diversity. Scand J Gastroenterol 43: 831–841. 18584522 10.1080/00365520801935434

[5] Hamad I, Sokhna C, Raoult D, Bittar F (2012) Molecular detection of eukaryotes in a single human stool sample from Senegal. PLoS One 7: e40888. 22808282 10.1371/journal.pone.0040888 PMC3396631

[6] Gow NA, Hube B (2012) Importance of the Candida albicans cell wall during commensalism and infection. Curr Opin Microbiol 15: 406–412. 22609181 10.1016/j.mib.2012.04.005

[7] Miranda LN, van der Heijden IM, Costa SF, Sousa AP, Sienra RA, et al (2009) Candida colonisation as a source for candidaemia. J Hosp Infect 72: 9–16. 19303662 10.1016/j.jhin.2009.02.009

[8] Nucci M, Anaissie E (2001) Revisiting the source of candidemia: skin or gut? Clin Infect Dis 33: 1959–1967. 11702290 10.1086/323759

[9] Koh AY, Kohler JR, Coggshall KT, Van Rooijen N, Pier GB (2008) Mucosal damage and neutropenia are required for Candida albicans dissemination. PLoS Pathog 4: e35. 18282097 10.1371/journal.ppat.0040035 PMC2242836

[10] Li Q, Wang C, Tang C, He Q, Li N, et al. (2013) Dysbiosis of Gut Fungal Microbiota is Associated With Mucosal Inflammation in Crohn's Disease. J Clin Gastroenterol. 10.1097/MCG.0000000000000035 PMC4059552 24275714

[11] Herre J, Marshall AS, Caron E, Edwards AD, Williams DL, et al (2004) Dectin-1 uses novel mechanisms for yeast phagocytosis in macrophages. Blood 104: 4038–4045. 15304394 10.1182/blood-2004-03-1140

[12] Vautier S, Drummond RA, Redelinghuys P, Murray GI, MacCallum DM, et al (2012) Dectin-1 is not required for controlling Candida albicans colonization of the gastrointestinal tract. Infect Immun 80: 4216–4222. 22988015 10.1128/IAI.00559-12 PMC3497410

[13] Franke A, McGovern DP, Barrett JC, Wang K, Radford-Smith GL, et al (2010) Genome-wide meta-analysis increases to 71 the number of confirmed Crohn's disease susceptibility loci. Nature genetics 42: 1118–1125. 21102463 10.1038/ng.717 PMC3299551

[14] McGovern DP, Gardet A, Torkvist L, Goyette P, Essers J, et al (2010) Genome-wide association identifies multiple ulcerative colitis susceptibility loci. Nature genetics 42: 332–337. 20228799 10.1038/ng.549 PMC3087600

[15] Seow CH, Stempak JM, Xu W, Lan H, Griffiths AM, et al (2009) Novel anti-glycan antibodies related to inflammatory bowel disease diagnosis and phenotype. The American journal of gastroenterology 104: 1426–1434. 19491856 10.1038/ajg.2009.79

[16] Strijbis K, Tafesse FG, Fairn GD, Witte MD, Dougan SK, et al (2013) Bruton's Tyrosine Kinase (BTK) and Vav1 contribute to Dectin1-dependent phagocytosis of Candida albicans in macrophages. PLoS Pathog 9: e1003446. 23825946 10.1371/journal.ppat.1003446 PMC3694848

[17] Jongstra-Bilen J, Puig Cano A, Hasija M, Xiao H, Smith CI, et al (2008) Dual functions of Bruton's tyrosine kinase and Tec kinase during Fcgamma receptor-induced signaling and phagocytosis. J Immunol 181: 288–298. 18566394 10.4049/jimmunol.181.1.288

[18] Khan WN, Alt FW, Gerstein RM, Malynn BA, Larsson I, et al (1995) Defective B cell development and function in Btk-deficient mice. Immunity 3: 283–299. 7552994 10.1016/1074-7613(95)90114-0

[19] Kerner JD, Appleby MW, Mohr RN, Chien S, Rawlings DJ, et al (1995) Impaired expansion of mouse B cell progenitors lacking Btk. Immunity 3: 301–312. 7552995 10.1016/1074-7613(95)90115-9

[20] White SJ, Rosenbach A, Lephart P, Nguyen D, Benjamin A, et al (2007) Self-regulation of Candida albicans population size during GI colonization. PLoS Pathog 3: e184. 18069889 10.1371/journal.ppat.0030184 PMC2134954

[21] Reeves PG, Nielsen FH, Fahey GC Jr (1993) AIN-93 purified diets for laboratory rodents: final report of the American Institute of Nutrition ad hoc writing committee on the reformulation of the AIN-76A rodent diet. J Nutr 123: 1939–1951. 8229312 10.1093/jn/123.11.1939

[22] Brown DH Jr, Giusani AD, Chen X, Kumamoto CA (1999) Filamentous growth of Candida albicans in response to physical environmental cues and its regulation by the unique CZF1 gene. Mol Microbiol 34: 651–662. 10564506 10.1046/j.1365-2958.1999.01619.x

[23] Jawhara S, Thuru X, Standaert-Vitse A, Jouault T, Mordon S, et al (2008) Colonization of mice by Candida albicans is promoted by chemically induced colitis and augments inflammatory responses through galectin-3. The Journal of infectious diseases 197: 972–980. 18419533 10.1086/528990

[24] Jawhara S, Mogensen E, Maggiotto F, Fradin C, Sarazin A, et al (2012) Murine model of dextran sulfate sodium-induced colitis reveals Candida glabrata virulence and contribution of beta-mannosyltransferases. J Biol Chem 287: 11313–11324. 22291009 10.1074/jbc.M111.329300 PMC3322808

[25] de Hoon MJ, Imoto S, Nolan J, Miyano S (2004) Open source clustering software. Bioinformatics 20: 1453–1454. 14871861 10.1093/bioinformatics/bth078

[26] Saldanha AJ (2004) Java Treeview—extensible visualization of microarray data. Bioinformatics 20: 3246–3248. 15180930 10.1093/bioinformatics/bth349

[27] Gantner BN, Simmons RM, Underhill DM (2005) Dectin-1 mediates macrophage recognition of Candida albicans yeast but not filaments. EMBO J 24: 1277–1286. 15729357 10.1038/sj.emboj.7600594 PMC556398

[28] Pierce JV, Kumamoto CA (2012) Variation in Candida albicans EFG1 expression enables host-dependent changes in colonizing fungal populations. MBio 3: e00117–00112. 22829676 10.1128/mBio.00117-12 PMC3413400

[29] Wheeler RT, Kombe D, Agarwala SD, Fink GR (2008) Dynamic, morphotype-specific Candida albicans beta-glucan exposure during infection and drug treatment. PLoS pathogens 4: e1000227. 19057660 10.1371/journal.ppat.1000227 PMC2587227

[30] Heinsbroek SE, Oei A, Roelofs JJ, Dhawan S, te Velde A, et al (2012) Genetic deletion of dectin-1 does not affect the course of murine experimental colitis. BMC Gastroenterol 12: 33. 22507600 10.1186/1471-230X-12-33 PMC3353241

[31] Shulzhenko N, Morgun A, Hsiao W, Battle M, Yao M, et al (2011) Crosstalk between B lymphocytes, microbiota and the intestinal epithelium governs immunity versus metabolism in the gut. Nat Med 17: 1585–1593. 22101768 10.1038/nm.2505 PMC3902046

[32] Dieleman LA, Ridwan BU, Tennyson GS, Beagley KW, Bucy RP, et al (1994) Dextran sulfate sodium-induced colitis occurs in severe combined immunodeficient mice. Gastroenterology 107: 1643–1652. 7958674 10.1016/0016-5085(94)90803-6

[33] Mangla A, Khare A, Vineeth V, Panday NN, Mukhopadhyay A, et al (2004) Pleiotropic consequences of Bruton tyrosine kinase deficiency in myeloid lineages lead to poor inflammatory responses. Blood 104: 1191–1197. 15117762 10.1182/blood-2004-01-0207

[34] Mukhopadhyay S, George A, Bal V, Ravindran B, Rath S (1999) Bruton's tyrosine kinase deficiency in macrophages inhibits nitric oxide generation leading to enhancement of IL-12 induction. J Immunol 163: 1786–1792. 10438910

[35] Honda F, Kano H, Kanegane H, Nonoyama S, Kim ES, et al (2012) The kinase Btk negatively regulates the production of reactive oxygen species and stimulation-induced apoptosis in human neutrophils. Nat Immunol 13: 369–378. 22366891 10.1038/ni.2234

[36] Li MO, Wan YY, Flavell RA (2007) T cell-produced transforming growth factor-beta1 controls T cell tolerance and regulates Th1- and Th17-cell differentiation. Immunity 26: 579–591. 17481928 10.1016/j.immuni.2007.03.014

[37] Travis MA, Reizis B, Melton AC, Masteller E, Tang Q, et al (2007) Loss of integrin alpha(v)beta8 on dendritic cells causes autoimmunity and colitis in mice. Nature 449: 361–365. 17694047 10.1038/nature06110 PMC2670239

[38] Gorelik L, Flavell RA (2000) Abrogation of TGFbeta signaling in T cells leads to spontaneous T cell differentiation and autoimmune disease. Immunity 12: 171–181. 10714683 10.1016/s1074-7613(00)80170-3

[39] Olszak T, Neves JF, Dowds CM, Baker K, Glickman J, et al. (2014) Protective mucosal immunity mediated by epithelial CD1d and IL-10. Nature. 10.1038/nature13150 PMC4132962 24717441

[40] Harrington LE, Hatton RD, Mangan PR, Turner H, Murphy TL, et al (2005) Interleukin 17-producing CD4+ effector T cells develop via a lineage distinct from the T helper type 1 and 2 lineages. Nat Immunol 6: 1123–1132. 16200070 10.1038/ni1254

[41] Park H, Li Z, Yang XO, Chang SH, Nurieva R, et al (2005) A distinct lineage of CD4 T cells regulates tissue inflammation by producing interleukin 17. Nat Immunol 6: 1133–1141. 16200068 10.1038/ni1261 PMC1618871

[42] Katayama M, Ohmura K, Yukawa N, Terao C, Hashimoto M, et al (2013) Neutrophils are essential as a source of IL-17 in the effector phase of arthritis. PLoS One 8: e62231. 23671588 10.1371/journal.pone.0062231 PMC3646022

[43] Gladiator A, Wangler N, Trautwein-Weidner K, LeibundGut-Landmann S (2013) Cutting edge: IL-17-secreting innate lymphoid cells are essential for host defense against fungal infection. J Immunol 190: 521–525. 23255360 10.4049/jimmunol.1202924

[44] Monteleone I, Sarra M, Pallone F, Monteleone G (2012) Th17-related cytokines in inflammatory bowel diseases: friends or foes? Curr Mol Med 12: 592–597. 22515978 10.2174/156652412800620066

[45] Jawhara S, Habib K, Maggiotto F, Pignede G, Vandekerckove P, et al (2012) Modulation of intestinal inflammation by yeasts and cell wall extracts: strain dependence and unexpected anti-inflammatory role of glucan fractions. PLoS One 7: e40648. 22848391 10.1371/journal.pone.0040648 PMC3407157

[46] Mason KL (2012) Erb Downward JR (2012) Mason KD, Falkowski NR, Eaton KA, et al (2012) Candida albicans and bacterial microbiota interactions in the cecum during recolonization following broad-spectrum antibiotic therapy. Infect Immun 80: 3371–3380. 22778094 10.1128/IAI.00449-12 PMC3457555

[47] Erb Downward JR (2013) Falkowski NR, Mason KL, Muraglia R, Huffnagle GB (2013) Modulation of post-antibiotic bacterial community reassembly and host response by Candida albicans. Sci Rep 3: 2191. 23846617 10.1038/srep02191 PMC3709164

[48] Round JL, Mazmanian SK (2010) Inducible Foxp3+ regulatory T-cell development by a commensal bacterium of the intestinal microbiota. Proc Natl Acad Sci U S A 107: 12204–12209. 20566854 10.1073/pnas.0909122107 PMC2901479

